# Quinine-Resistant Malaria in Traveler Returning from Senegal, 2007

**DOI:** 10.3201/eid1603.091669

**Published:** 2010-03

**Authors:** Bruno Pradines, Thierry Pistone, Khaled Ezzedine, Sébastien Briolant, Lionel Bertaux, Marie-Catherine Receveur, Daniel Parzy, Pascal Millet, Christophe Rogier, Denis Malvy

**Affiliations:** Institut de Médecine Tropicale du Service de Santé des Armées, Marseille, France (B. Pradines, S. Briolant, L. Bertaux, D. Parzy, C. Rogier); Centre Hospitalier Universitaire St.-André, Bordeaux, France (T. Pistone, K. Ezzedine, M.-C. Receveur, D. Malvy); Université Victor Segalen Bordeaux 2, Bordeaux (K. Ezzedine, P. Millet, D.Malvy)

**Keywords:** Quinine, drug resistance, malaria, parasites, Plasmodium falciparum, Senegal, France, traveler, dispatch

## Abstract

We describe clinical and parasitologic features of in vivo and in vitro *Plasmodium falciparum* resistance to quinine in a nonimmune traveler who returned to France from Senegal in 2007 with severe imported malaria. Clinical quinine failure was associated with a 50% inhibitory concentration of 829 nmol/L. Increased vigilance is required during treatment follow-up.

Resistance of *Plasmodium falciparum* to antimalarial drugs is one the most worrisome problems in tropical medicine. Quinine remains the first-line antimalarial option for treatment of patients with complicated malaria in Europe and Africa. However, emergence of quinine resistance has been sparsely documented (*1*). Maximizing the efficacy and longevity of quinine as a drug to control malaria will critically depend on pursuing intensive research into identifying in vitro markers and implementing active in vitro and in vivo surveillance programs such as those supported by the World Antimalarial Resistance Network. Such molecular markers are needed to monitor temporal trends in parasite susceptibility (*2*). We report quinine-resistant *P.* *falciparum* malaria in a patient who returned to France from Senegal.

## The Patient

A 17-year-old white man from France spent ≈2 months (April and most of May) in 2007 in Dielmo, Senegal, where malaria is highly endemic and shows intense perennial transmission (*3*). He did not use antimalarial prophylaxis or protection against mosquitoes. After returning to France, he was admitted to the Bordeaux University Hospital Center on May 27, 2007 (day 0). The patient had *P*. *falciparum* parasitemia level of 7% and a 2-day history of fever, myalgia, vomiting, and rapid deterioration of consciousness into an arousable coma. A diagnosis of severe malaria with cerebral involvement was confirmed.

Intravenous quinine formiate (loading dose 17 mg/kg) was administered, followed by a maintenance dose (8.3 mg/kg 3×/day for 7 days). The patient was afebrile on day 3, and his thin and thick blood films became negative for *P*. *falciparum* on day 6. He was discharged from the hospital on day 7. However, on day 26, he relapsed and had fever and vomiting. He was hospitalized again on day 27 with a core temperature of 40°7C, deterioration of consciousness, and a *P*. *falciparum* parasitemia level of 4%. He received the same regimen of quinine formiate plus intravenous clindamycin (10 mg 3×/day) for 7 days. His serum quinine level (free and bound drug assayed by high performance liquid chromatography) taken immediately before the fourth drug dose was low (7 mg/L). The patient was then given quinine (10 mg/kg 3×/day from day 30 through day 34). Serum quinine levels then increased and fever cleared within 72 hours. However, a blood smear was positive on day 34.

Because of the treatment failure with quinine and clindamycin, the patient was treated with oral co-artemether (20 mg artemether and 120 mg lumefantrine, given as 4 tablets, followed by 4 tablets after 8 hours, and 4 tablets 2×/day for 2 days; total = 24 tablets). Parasitic clearance was observed within 48 hours. Blood smears and results of a PCR for *P*. *falciparum* were negative from day 36 through day 62. No further recrudescence occurred over the next 12 months.

The isotopic microdrug susceptibility tests used have been described (*4*). The chloroquine-susceptible 3D7 *P*. *falciparum* clone (Africa) and the chloroquine-resistant W2 clone (Indochina), after 2 rounds of sorbitol synchronization, were used as controls. The 50% inhibitory concentration (IC_50_) values for 12 antimalarial drugs for the study isolate and these 2 controls are shown in the Table. The strain isolated on day 27 showed reduced susceptibility to quinine (IC_50_ 829 nmol/L, threshold 800 nmol/L) and chloroquine (472 nmol/L, threshold 100 nmol/L). The IC_50_ for clindamycin was 39 μmol/L (the in vitro resistance cutoff value was not determined). The isolate was susceptible to all other antimalarial drugs tested. Phenotypes and genotypes were assessed only for parasites obtained on day 27.

**Table:** In vitro susceptibility of 3 *Plasmodium falciparum* isolates to 12 antimalarial drugs, France*

Drug	50% Inhibitory concentration			
	Study isolate	3D7	W2	Cutoff value
Quinine	829 nmol/L	157 nmol/L	574 nmol/L	>800 nmol/L
Chloroquine	472 nmol/L	21 nmol/L	392 nmol/L	>100 nmol/L
Mefloquine	10.4 nmol/L	49.3 nmol/L	39.3 nmol/L	>30 nmol/L
Lumefantrine	19 nmol/L	29 nmol/L	35 nmol/L	>150 nmol/L
Monodesethylamodiaquine	47 nmol/L	17 nmol/L	162 nmol/L	>80 nmol/L
Dihydroartemisinin	1.1 nmol/L	2.5 nmol/L	3.0 nmol/L	>10.5 nmol/L
Atovaquone	13.3 nmol/L	4.1 nmol/L	3.6 nmol/L	>350 nmol/L
Cycloguanil	70 nmol/L	<10 nmol/L	1191 nmol/L	>500 nmol/L
Pyrimethamine	354 nmol/L	<50 nmol/L	9139 nmol/L	>2,000 nmol/L
Doxycycline	12.8 μmol/L	10.5 μmol/L	13.5 μmol/L	>35 μmol/L
Azithromycin	24 μmol/L	48 μmol/L	39 μmol/L	ND
Clindamycin	39 μmol/L	108 μmol/L	126 μmol/L	ND

*P. *falciparum* strains 3D7 and W2 were used as controls. ND, not determined.

We concurrently screened blood samples for resistance-associated point mutations. A sequence containing the ms4760 microsatellite was amplified as described (*5*). The observed ms4760–18 profile was composed of 2 DNNND repeats and 2 DDDNHNDNHNN repeats. Genotyping of the *P*. *falciparum* chloroquine resistance transporter (*Pfcrt*) gene, which encodes a transport protein involved in chloroquine resistance (K76T), and the dihydroopteroate synthase gene, which encodes the sulfadoxine target (A437G), identified the resistant allele in our isolate (*6*). There was no mutation in codon 268, which encodes the atovaquone target (*4*). The isolate had only 1 copy of the *P*. *falciparum* multidrug resistance (*Pfmdr1*) gene and a mutation in codon 184, which suggested in vitro susceptibility to mefloquine (*7*). Amplification of DNA from parasites obtained on day 0 and preserved on fixed and stained thin blood films by a modification of the procedure of Edoh et al. (*8*) was not successful.

## Conclusions

Quinine remains a reliable treatment for patients with complicated or severe *P*. *falciparum* malaria outside southern Asia. Clinical failure with quinine used alone or in combination with clindamycin is common in Africa. In our case-patient, a correlation between the results of the in vivo and in vitro assessments was demonstrated at day 27. Because of the lack of reliable data on the correlation between quinine IC_50_ and clinical failure, arbitrary IC_50_ cutoff values were chosen for in vitro quinine resistance (300 nmol/L, 500 nmol/L, or 800 nmol/L) (*9*).

Quinine resistance appears to share common characteristics with chloroquine resistance. It is associated with mutations in the *pfmdr1* (*10*) and *pfcrt* (*11*) genes. Nevertheless, the mechanism of quinine resistance is still unknown. In addition to the *pfmdr1* and *pfcrt* genes, other genetic polymorphisms such as microsatellite length variations in the *P*. *falciparum* sodium/hydrogen exchanger (*pfnhe-1*) gene (*5*) and mutations in the *P*. *falciparum* multidrug resistance protein gene may contribute to quinine resistance (*12*).

We report an association of clinical failure of quinine treatment with an IC_50_ of 829 nmol/L, a mutation in codon 76 of the *pfcrt* gene, and an ms4760–18 profile for *pfnhe-1* composed of 2 DNNND repeats. Isolates of *P*. *falciparum* with >2 DNNND repeats may be associated with reduced susceptibility to quinine. Henry et al. (*5*) reported that 2 DNNND repeats were associated with quinine IC_50_ values ranging from 300 nmol/L to 700 nmol/L, and that 3 repeats were associated with an IC_50_ >600 nmol/L. However, the 3 strains with IC_50_s >800 nmol/L had >2 DNNND repeats (*6*). Our results are consistent with these data.

*P*. *falciparum* resistance levels may differ depending on malaria transmission and drug pressure. Data from Senegal are fragmentary and were obtained by in vitro susceptibility studies conducted with isolates reported to have decreased in vitro susceptibility to quinine (*6*). Our patient had traveled to Dielmo, Senegal, where in vitro surveillance of antimalarial drug susceptibility has been conducted since 1996. During 1996–2005, the overall prevalence of isolates with IC_50_ >800 nmol/L for quinine was <6%: 1% in 1996, 4% in 1997, 0% in 1998, 6% in 1999, and 0% in 2005 (*13*). Quinine was used for 96.4% of the treatments administered in Dielmo during 1990–1995 (*14*). This drug has since been replaced by chloroquine, sulfadoxine-pyrimethamine, and artemisinin-based combination therapies.

We report a patient with clinical failure associated quinine resistance in a traveler to Senegal. Our results are consistent with those of a recent review of the Uganda Malaria Surveillance Project that reported a higher risk for selecting quinine-resistant parasites associated with a 7-day quinine treatment course (*15*). Thus, resistance to quinine should be monitored in West Africa. Although such clinical failure of therapy is rare, increased vigilance is required during treatment follow-up, and surveillance of the parasite population should also be increased.
